# Acute phase reactants in paediatric chronic nonbacterial osteomyelitis: implications for diagnosis and correlation with disease phenotype

**DOI:** 10.1093/rheumatology/keag495

**Published:** 2026-09-09

**Authors:** Büşra Tetik Dinçer, Pınar Özge Avar-Aydın, Fatma Aydın, Doğacan Sarısoy, Elif Erorhan, Onur Bahçeci, Zeynep Birsin Özçakar

**Affiliations:** Department of Paediatric Rheumatology, Ankara University School of Medicine, Ankara, Türkiye; Department of Paediatric Rheumatology, Ankara University School of Medicine, Ankara, Türkiye; Department of Paediatric Rheumatology, Ankara University School of Medicine, Ankara, Türkiye; Department of Paediatric Rheumatology, Ankara University School of Medicine, Ankara, Türkiye; Department of Paediatric Rheumatology, Ankara University School of Medicine, Ankara, Türkiye; Department of Paediatric Rheumatology, Ankara University School of Medicine, Ankara, Türkiye; Department of Paediatric Rheumatology, Ankara University School of Medicine, Ankara, Türkiye

**Keywords:** acute phase reactants, chronic nonbacterial osteomyelitis, autoinflammatory bone disease, classification criteria

## Abstract

**Objectives:**

The role of acute phase reactants (APRs) in the diagnosis of chronic nonbacterial osteomyelitis (CNO) is controversial. Many patients have moderate elevation and some have normal levels. Markedly elevated APRs are more likely to have CNO mimickers that cause lower classification scores. This study aims to investigate how elevated APRs affect the classification of CNO at the diagnosis and their relationship with patient and disease characteristics.

**Methods:**

This retrospective single-centre study included patients diagnosed with CNO who were grouped according to APRs at diagnosis: markedly elevated APRs (CRP ≥30 mg/l and/or ESR ≥60 mm/h as proposed by classification criteria) and normal-to-moderately elevated APRs. Clinical and imaging findings, and treatment modalities were compared between groups.

**Results:**

Fifty-one patients with CNO were included into the study; 31% of them (*n* = 16) were classified in markedly elevated APRs group. They exhibited lower classification scores and haemoglobin, higher platelet and ferritin concentrations, more extensive involvement of CNO, and a higher likelihood for CNO-associated diseases. Median total number of bone lesions was significantly higher in this group, and a moderate correlation was observed between number of bone lesions and CRP. Pelvic bone, clavicle and sacroiliac joint involvement were significantly more common in patients with markedly elevated APRs.

**Conclusion:**

Markedly elevated APRs are observed in a substantial subset of CNO patients that may cause classification limitations. These patients show more severe systemic inflammation, more extensive involvement, particularly in clavicle, pelvis and sacroiliac joints, and a higher likelihood of CNO-associated diseases.

*Rheumatology* key messages

- Markedly elevated APRs are observed in a substantial subset of paediatric CNO patients, that may cause classification limitations.
- These patients show more severe systemic inflammation, more extensive involvement, particularly in clavicle, pelvis, and sacroiliac joints.
- These patients show more higher likelihood of CNO-associated diseases such as familial Mediterranean fever and inflammatory bowel disease.

## Introduction

Chronic nonbacterial osteomyelitis (CNO) is an autoinflammatory bone disease characterized by sterile bone inflammation [1]. It has a wide clinical spectrum of presentation from single site bone involvement lasting for a short period to multifocal chronic involvement with recurrent courses. Typical manifestations of CNO are bone pain and musculoskeletal functional limitation with an onset in late childhood to adolescence. CNO is associated with chronic inflammatory diseases such as cutaneous inflammatory disorders, inflammatory bowel disease (IBD) and spondyloarthropathies [2, 3].

There are no validated diagnostic criteria making CNO a diagnosis of exclusion [4, 5]. The insidious onset, variable clinical expression, imaging findings and limited clinical research result in diagnostic challenges and difficulties. A wide variety of disease mimickers including but not restricted to infection, malignancy and metabolic disorders need to be carefully evaluated. The lack of clues for these diseases [fever, cytopenias, highly elevated acute phase reactants (APRs), no response to antimicrobial treatment] supports the diagnosis of CNO with typical imaging findings on multifocal bones within typical locations such as clavicle and mandible. Bone biopsy may be still obtained although histological findings are also not specific for CNO [4, 6].

The disease is driven by a cytokine imbalance that promotes osteoclast activation and leads to sterile bone inflammation. Increasing evidence indicates a genetic predisposition involving inflammasome activation and IL-1 mediated pathways [1]. These findings support an autoinflammatory mechanism and suggest that the acute phase response maybe directly influenced by this underlying inflammatory process. In 2025, the first classification criteria of paediatric CNO were developed with a combination of international expert consensus and data-driven methods by the European Alliance of Associations for Rheumatology/American College of Rheumatology (EULAR/ACR) and further validated. APRs were included in the set of criteria [7, 8].

Acute-phase reactants, including C-reactive protein (CRP) and erythrocyte sedimentation rate (ESR), may be elevated and can serve as indicators of inflammatory activity in CNO. Normal or mildly elevated CRP and ESR are expected in typical CNO cases. Therefore, the 2025 EULAR/ACR paediatric CNO classification criteria proposed that CRP ≥30 mg/l and ESR ≥60 mm/h are unfavourable for CNO, resulting in a score of 0 from these two items [7]. However, the proportion of cases presenting with elevated APRs at diagnosis shows considerable variability [5, 9–11]. The relationship between APRs and disease severity, radiological lesion burden, extraosseous involvement and long-term outcomes remains unclear.

The aim of this study was to evaluate the APRs at diagnosis in patients with CNO, and to assess the frequency and level of elevated APRs responses. We also investigated how elevated APRs affect CNO classification at diagnosis according to the 2025 EULAR/ACR paediatric CNO classification criteria. Furthermore, we intended to evaluate the relationship between high APRs detected at diagnosis and patient and disease characteristics.

## Methods

### Study design and population

This study is a retrospective, observational, single-centre study evaluating data from patients diagnosed with CNO who were followed up at the Department of Paediatric Rheumatology between January 2016 and January 2026. The diagnosis of CNO was based on clinical symptoms and imaging findings after excluding other diseases. Patients with a follow-up of <6 months (*n* = 2) were excluded from the study.

The patients’ age, sex, age at diagnosis, time to diagnosis, clinical findings, laboratory results, bone biopsy, radiological imaging findings and treatment regimens were retrospectively obtained from the electronic medical records. The standard deviation scores (SDS) of the height and body weight during the visits were evaluated according to the age- and sex-specific growth references for Turkish children [12]. All patients were diagnosed with CNO based on clinical and radiological findings after exclusion of malignancy and infection, using the criteria defined by Jansson *et al.* [13]. In addition, the 2025 ACR/EULAR paediatric CNO classification criteria were applied for patient classification, and total scores were calculated accordingly [7].

### Radiological evaluation

Imaging modalities used in the study population including regional magnetic resonance imaging (MRI), whole-body MRI (WBMRI) and bone scintigraphy were reviewed to characterize bone lesions. At our centre, patients with typical CNO lesions on regional MRIs underwent WBMRI to identify multifocal involvement. Prior to 2020, when WBMRI was not available, bone scintigraphy was used for the same purpose.

Regional MRI examinations used pre-contrast three-plane T1-weighted imaging, coronal STIR, axial fat-suppressed T2-weighted sequences and diffusion-weighted imaging. Where necessary, post-contrast fat-suppressed three-plane T1-weighted images were obtained to complete the evaluation.

Whole-body MRI was performed using a 1.5 Tesla MR device (Achieva, Philips Healthcare, Best, Netherlands). The coronal plane was primarily preferred in the imaging protocol. In addition, the spine and ankles were evaluated in the sagittal plane, while the pelvis and knee joints were evaluated in the axial plane. The coronal plane was used as the main imaging plane because it covers a wide anatomical area and allows for detailed examination of the axial skeleton. Additional sagittal and axial sections were obtained when necessary. The WBMRI protocol included pre-contrast sagittal T1-weighted, coronal T1-weighted and coronal STIR sequences. Diffusion-weighted imaging (b: 0 and 1000 s/mm²) was also performed in the axial plane. Contrast-enhanced coronal and sagittal T1-weighted images were acquired in patients with suspicious signal changes on pre-contrast images.

Based on WBMRI findings, patients with a single bone lesion were classified as ‘*unifocal*’, and those with multiple bone lesions were classified as ‘*multifocal*’. The presence of bilateral involvement in at least one bone was defined as ‘*symmetrical involvement*’. ‘*Total number of bone lesions*’ on WBMRI referred to the sum of CNO-related inflammatory bone lesions on WBMRI. All MRI images were evaluated by a single paediatric radiologist with >20 years of experience in musculoskeletal MRI.

### Study groups according to acute phase reactants

Patients with ESR ≥60 mm/h and/or CRP ≥ 30 mg/l at the diagnosis were defined as the ‘*patients with markedly elevated APRs*’ group according to the threshold values specified in the 2025 ACR/EULAR paediatric CNO classification criteria while patients below these thresholds were classified as ‘*patients with normal-to-moderately elevated APRs*’ [7]. Further, patients with any elevation in CRP and/or ESR at the diagnosis of CNO were defined as ‘*patients with high APRs*’ while others were ‘*patients with normal APRs*’. Normal ranges for CRP and ESR were <5 mg/l and <20 mm/h, respectively.

‘Patients with markedly elevated APRs’ and ‘patients with normal-to-moderately elevated APRs’, and ‘patients with high APRs’ and ‘patients with normal APRs’ were compared in terms of demographics, clinical and imaging findings, CNO-associated diseases and treatment modalities.

### CNO-associated diseases

CNO-associated diseases included familial Mediterranean fever (FMF), IBD, spondyloarthropathies, psoriasis, juvenile idiopathic arthritis (JIA) and uveitis in the study population. The diagnosis of FMF was made according to the Yalçınkaya criteria [14], and the *MEFV* gene analysis were evaluated. The *MEFV* gene analysis was performed using massively parallel sequencing (MPS). All exons of the *MEFV* gene were sequenced. The pathogenicity of the sequence variants was classified accordingly [15]. At our clinic, the indications of the *MEFV* gene testing for the patients with CNO include: the presence of symptoms or family history of FMF, persistent elevation of APRs and extensive involvement of CNO. JIA was classified using the International League of Associations for Rheumatology (ILAR) criteria [16]. IBD was diagnosed by a paediatric gastroenterologist according to the European Society for Paediatric Gastroenterology, Hepatology and Nutrition/Paediatric IBD Porto Group (ESPGHAN/Porto) criteria [17]. Psoriasis was diagnosed clinically based on characteristic dermatological findings confirmed by a dermatologist.

### Disease course

‘*Persistent disease*’ was defined as the absence of both clinical and radiological remission after 12 months of treatment, ‘*Clinical remission*’ required complete resolution of symptoms for a minimum of six consecutive months, while ‘*radiological remission*’ was defined by the absence of active inflammatory bone lesions on WBMRI.

### Statistical analysis

Statistical analyses were performed using IBM SPSS Statistics 25.0 software. The distribution of continuous variables was assessed using the Shapiro–Wilk test. Data showing a normal distribution were presented as mean ± standard deviation (SD), while data not showing a normal distribution were presented as median [interquartile range (IQR), 25th–75th percentile (25–75%)]. Categorical variables were expressed as numbers and percentages.

For comparisons between two groups, the independent samples *t* test was used for continuous variables showing a normal distribution, and the Mann–Whitney *U* test was used for variables not showing a normal distribution. The χ^2^ or Fisher’s exact test was applied for the comparison of categorical variables. Correlation between APRs and total number of bone lesions on WBMRI was evaluated by Spearman coefficients (r), whereby r ≥ 0.2 was considered significant, 0.4–0.6 as moderate and r ≥ 0.6 as strong. A two-sided *P*-value <0.05 was considered statistically significant.

### Ethical approval

The study was approved by the local ethics committee of the institution (24.03.2026; reference number: İ03-306–26). Due to the retrospective design of the study, informed consent was waived from patients and parents.

## Results

### Study population

A total of 51 patients with CNO were included in the study. The median age at diagnosis was 11 years (8–14), and 33 patients (64.7%) were male. The median time from symptom onset to diagnosis was 8 (4–17) months and the median follow-up duration was 40 (21.5–64) months.

The median total score of the 2025 ACR/EULAR paediatric CNO classification criteria was found to be 67 (65–76). Five patients had a score of <55 according to the 2025 ACR/EULAR paediatric CNO classification criteria; four of these had higher APRs than the established cut-offs of CRP and ESR, while one patient could not meet the required score to be classified as CNO due to unifocal CNO lesion in the foot.

Bone biopsy was performed in 11 patients (21.6%). Three of them had markedly elevated APRs. Median CRP was 7.2 mg/l (IQR 2.9–29) and median ESR was 24 mm/h (IQR 12–59) in patients who underwent bone biopsy.

All patients received non-steroidal anti-inflammatory drugs (NSAIDs) as the first-line treatment. Corticosteroids were needed in 21.6%, while conventional disease-modifying antirheumatic drugs (cDMARDs) were used in 47.1% and biologic DMARDs (bDMARDs) in 43.1% of the study population. Radiological remission could not be evaluated in two patients due to the absence of WBMRI in the follow-up. Six patients had pathological vertebral fractures detected by imaging.

Thirty-five patients (68.6%) were classified in the ‘patients with normal-to-moderately elevated APRs’ group, whereas 16 (31.4%) were within the ‘patients with markedly elevated APRs’ group. While 19 patients had normal APRs and 32 had increased APRs. Nine patients with elevated CRP had normal ESR, whereas three patients with elevated ESR were found to have normal CRP.

### Clinical findings and treatments according to APRs

The comparison of the demographic, anthropometric and clinical characteristics between study groups are presented in Table 1. When comparing patients’ symptoms, only the presence of fever was significantly more common in patients with markedly elevated APRs compared with the patients with normal-to-moderately elevated APRs (2.9% *vs* 31.3%, *P* = 0.003).

**Table 1:** Demographic, anthropometric and clinical characteristics of the study groups.

Variables	All patients (N = 51)	Patients with normal-to-moderately elevated APRs (N = 35)	Patients with markedly elevated APRs (N = 16)	p1-value*	Patients with normal APRs (N = 19)	Patients with high APRs (N = 32)	p2-value**
**Age at diagnosis [years, median (IQR)]**	11 (9-14)	11 (9-13)	11 (10-14)	*0.442^a^*	11 (9-13)	11 (10-14)	*0.550^a^*
**Sex [*n* (%)]**				*0.824^b^*			*0.433^b^*
Female	18 (35.3%)	12 (34.3%)	6 (37.5%)		8 (42.1%)	10 (31.3%)	
Male	33 (64.7%)	23 (65.7%)	10 (62.5%)		11 (57.9%)	22 (68.8%)	
**Time to diagnosis [months, median (IQR)]**	8 (4–17)	8 (3–20)	8.5 (5.5–14.3)	*0.823^a^*	8 (3–14)	8.5 (5.5–20.3)	*0.494 ^a^*
**Follow-up [months, median (IQR)]**	40 (21.5–64)	39 (21–59)	45.5 (26.5–67.8)	*0.490^a^*	27 (19–55)	45 (27.8–63.5)	*0.279 ^a^*
**Weight SDS [median (IQR)]**	0.09 (–1.10–0.94)	0.13 (–1.05–0.94)	–0.02 (–1.10–0.87)	*0.917^a^*	0.34 (–0.603–1.48)	–0.43 (–1.52–0.79)	*0.154 ^a^*
**Height SDS [median (IQR)]**	0.13 (–0.67–1.26)	–0.03 (–0.88–1.18)	0.61 (–0.27–1.43)	*0.294^a^*	0.275 (–0.505–1.18)	0.06 (–0.745–1.43)	*0.848^a^*
**BMI SDS [median (IQR)]**	–0.16 (–1.14–0.603)	–0.16 (–1.14–0.98)	–0.22 (–0.89–0.31)	*0.585^a^*	0.37 (–0.613–1.23)	–0.415 (–1.22–0.318)	*0.077 ^a^*
**Exon 10 mutation in the *MEFV* gene [*n* = 37 (%)]**	11 (29.7%)	6 (25%)	5 (38.5%)	*0.392^b^*	2 (18.2%)	9 (34.6%)	*0.445^c^*
**HLA B27 positivity [*n* = 39 (%)]**	2 (5.1%)	1 (3.7%)	1 (8.3%)	*0.526^c^*	1 (7.1%)	1 (4%)	*1.00^c^*
**ANA positivity [*n* = 42 (%)]**	11 (26.2%)	8 (29.6%)	3 (20%)	*0.717^c^*	4 (28.6%)	7 (25%)	*1.00^c^*
**Total score of the 2025 ACR/EULAR paediatric CNO classification criteria [median (IQR)]**	67 (65–76)	72 (67–80)	63 (57–67)	***<0.001*** *^a^*	72 (66–79)	67 (61.3–74.5)	*0.132 ^a^*
**CNO-associated diseases [*n* (%)]**	22 (43.1%)	13 (37.1%)	9 (56.2%)	*0.177^c^*	5 (26.3%)	17 (53.1%)	*0.925^c^*
**Patients with FMF and/or IBD**	15 (29.4%)	8 (22.9%)	7 (43.8%)	*0.187^b^*	3 (15.8%)	12 (37.5%)	*0.123^c^*
**Treatments [*n* (%)]**							
**Oral corticosteroids**	11 (21.6%)	5 (14.3%)	6 (37.5%)	*0.061^b^*	4 (21.1%)	7 (21.9%)	*1.00^c^*
**cDMARD**	24 (47.1%)	16 (45.7%)	8 (50%)	*0.776^b^*	7 (36.8%)	17 (53.1%)	*0.260^b^*
**bDMARD**	22 (43.1%)	14 (40%)	8 (50%)	*0.503^b^*	5 (26.3%)	17 (53.1%)	*0.062^b^*
**Bisphosphonates**	7 (13.7%)	4 (11.4%)	3 (18.8%)	*0.664^c^*	1 (5.3%)	6 (18.8%)	*0.236^c^*
**Colchicine**	11 (29.7%)	6 (25%)	5 (38.5%)	*0.392^b^*	2 (18.2%)	9 (34.6%)	*0.445^c^*
**Clinical remission [*n* (%)]**	46 (90.2%)	32 (91.4)	14 (87.5%)	*0.643^c^*	18 (94.7%)	28 (87.5%)	*0.639^c^*
**Radiological remission [*n* (%)]**	32 (65.3%)	23 (67.6%)	9 (60%)	*0.604^b^*	13 (68.4%)	19 (63.3%)	*0.715^b^*

a Mann–Whitney *U* test,

b χ^2^ test,

c Fisher’s exact test.

* p1-value represents the comparison between groups: ‘patients with normal-to-moderately elevated APRs’ and ‘patients with markedly elevated APRs’. Patients with ESR ≥60 mm/h and/or CRP ≥ 30 mg/L at the diagnosis were defined as the ‘patients with markedly elevated APRs’ according to the threshold values specified in the 2025 ACR/EULAR paediatric CNO classification criteria while patients below these thresholds were classified as ‘patients with normal-to-moderately elevated APRs’.

** p2-value represents the comparison between groups: ‘patients with normal APRs’ and ‘patients with high APRs’. Any elevation in CRP and/or ESR at the diagnosis of CNO was defined as ‘patients with high APRs’ while others were ‘patients with normal APRs’.

ACR: American College of Rheumatology; ANA: antinuclear antibodies; APR: acute phase reactant; BMI: body mass index; bDMARD: biologic disease modifying antirheumatic drug; cDMARD: conventional disease-modifying antirheumatic drugs; CNO: chronic nonbacterial osteomyelitis; EULAR: European Alliance of Associations for Rheumatology; FMF: familial Mediterranean fever; HLA: human leucocyte antigen; IQR: interquartile range; SDS: standard deviation score.

The median total score of the 2025 ACR/EULAR paediatric CNO classification criteria was significantly lower in patients with markedly elevated APRs compared with others with normal-to-moderately elevated APRs [63 (57–67) *vs* 72 (67–80), *P* < 0.001].

The frequency of CNO-associated diseases and patients with FMF and/or IBD was higher in patients with markedly elevated APRs (56.2% *vs* 37.1%) and patients with high APRs (53.1% *vs* 26.3%) compared with other groups, although this difference was not statistically significant.

Anthropometric measurements, treatment modalities, and clinical and radiological remission rates were similar between the study groups.

### Laboratory findings according to APRs

Laboratory findings according to the study groups at the diagnosis of CNO are shown in Supplementary Table S1. In comparisons between the study groups, haemoglobin and mean platelet volume (MPV) were significantly lower (*P* = 0.049 and *P* = 0.002, respectively), whereas platelet count and serum ferritin were significantly higher (*P* = 0.003 and *P* = 0.041, respectively) in the patients with markedly elevated APRs group compared with the patients with normal-to-moderately elevated APRs.

When the patients with normal APRs and high APRs were compared, MPV was found to be significantly lower (*P* = 0.010), while white blood cell (WBC) count and red cell distribution width (RDW) were significantly higher in the patients with high APRs (*P* = 0.025 and *P* = 0.038, respectively).

### Radiologic findings according to APRs

When imaging characteristics were evaluated 96.1% of patients demonstrated multifocal and 78.4% showed symmetric involvement. Femur (58.8%) was the most commonly involved bone, followed by sacroiliac joint (47.1%) and tibia (45.1%) (Table 2). The median of total number of bone lesions on WBMRI was 7 (5–12), which was significantly higher in patients with markedly elevated APRs compared with patients with normal-to-moderately elevated APRs [12 (7–15) *vs* 6 (5–8), *P* = 0.006]. When the correlation of total number of bone lesions on WBMRI was evaluated, a moderate correlation was observed with CRP (r = 0.412, *P* = 0.003), whereby no significant correlation was present with ESR (r = 0.252, *P* = 0.075). Pelvis, clavicle and sacroiliac joint involvements were significantly more frequently detected in the patients with markedly elevated APRs compared with the patients with normal-to-moderately elevated APRs (all *P *< 0.05). Similarly, clavicle and sacroiliac joint involvement were significantly more common in the patients with high APRs compared with the patients with normal APR group (all *P* < 0.05).

**Table 2:** Imaging findings of the study groups.

Variables	All patients (N = 51)	Patients with normal-to-moderately elevated APRs (N = 35)	Patients with markedly elevated APRs (N = 16)	p1-value*	Patients with normal APRs (N = 19)	Patients with high APRs (N = 32)	p2-value**
**Total number of bone lesions [median (IQR)]**	7 (5-12)	6 (5-8)	12 (7-15)	***0.006*** *^a^*	6 (4-8)	8 (5-13)	*0.075^a^*
**MRI characteristics [n (%)]**							
**Multifocal pattern**	49 (96.1%)	33 (94.3)	16 (100%)	*1.000^b^*	18 (94.7%)	31 (96.9%)	*1.000^b^*
**Symmetry**	40 (78.4%)	27 (77.1%)	13 (81.3%)	*1.000^b^*	13 (68.4%)	27 (84.4%)	*0.180^c^*
**Sclerosis**	12 (23.5%)	9 (25.7%)	3 (18.8%)	*0.730^b^*	3 (15.8%)	9 (28.1%)	*0.497^b^*
**Periostal reaction**	7 (13.7%)	4 (11.4%)	3 (18.8%)	*0.664^b^*	2 (10.5%)	5 (15.6%)	*0.699^b^*
**Soft tissue oedema**	29 (56.9%)	19 (54.3%)	10 (62.5%)	*0.583^c^*	9 (47.4%)	20 (62.5%)	*0.291^b^*
**Lesion location [n (%)]**							
**Tibia**	23 (45.1%)	16 (45.7%)	7 (43.8%)	*0.896^c^*	9 (47.4%)	14 (43.8%)	*0.802^c^*
**Femur**	30 (58.8%)	18 (51.4%)	12 (75%)	*0.137^b^*	9 (47.4%)	21 (65.6%)	*0.200^c^*
**Pelvic bone**	20 (39.2%)	10 (28.6%)	10 (62.5%)	***0.021^c^***	5 (26.3%)	15 (46.9%)	*0.146^c^*
**Clavicle**	12 (23.5%)	5 (14.3%)	7 (43.8%)	***0.021^c^***	1 (5.3%)	11 (34.4%)	***0.020^b^***
**Mandible**	4 (7.8%)	3 (8.6%)	1 (6.3%)	*1.000^b^*	3 (15.8%)	1 (3.1%)	*0.140^b^*
**Skull**	2 (3.9%)	1 (2.9%)	1 (6.3%)	*0.533^b^*	1 (5.3%)	1 (3.1%)	*1.00^b^*
**Hand**	2 (3.9%)	1 (2.9%)	1 (6.3%)	*0.533^b^*	1 (5.3%)	1 (3.1%)	*1.00^b^*
**Sacroiliac joint**	24 (47.1%)	13 (37.1%)	11 (68.8%)	***0.036^c^***	4 (21.1%)	20 (62.5%)	***0.008^b^***
**Vertebra**	17 (33.3%)	9 (25.7%)	8 (50.0%)	*0.088^c^*	3 (15.8%)	14 (43.8%)	*0.065^b^*

a Mann-Whitney *U* test,

b Fisher’s exact test,

c χ^2^ test.

* p1-value represents the comparison between groups: ‘patients with normal-to-moderately elevated APRs’ and ‘patients with markedly elevated APRs’. Patients with ESR ≥60 mm/h and/or CRP ≥ 30 mg/L at the diagnosis were defined as the ‘patients with markedly elevated APRs’ according to the threshold values specified in the 2025 ACR/EULAR paediatric CNO classification criteria while patients below these thresholds were classified as ‘patients with normal-to-moderately elevated APRs’.

** p2-value represents the comparison between groups ‘patients with normal APRs’ and ‘patients with high APRs’. Any elevation in CRP and/or ESR at the diagnosis of CNO was defined as ‘patients with high APRs’ while others were ‘patients with normal APRs’.

APR: acute phase reactant, IQR: interquartile range, MRI: magnetic resonance imaging.

### Findings on CNO-associated diseases

Twenty-two patients (43.1%) had a CNO-associated disease. FMF was present in 11 patients (21.6%), IBD in five patients (9.8%), one patient had both FMF and IBD, uveitis in five patients (9.8%) and psoriasis in two patients (3.8%).

Exon 10 mutations in the *MEFV* gene were detected in 11 patients of the 37 patients who underwent *MEFV* gene testing. Two patients had homozygous *M694V* mutations, three had heterozygous *M694V* mutations, two had compound heterozygous *M694V/V726A* mutations, two had compound heterozygous *M694V/M680I* mutations, one had heterozygous *M680I* mutations and one had compound heterozygous *M680I/V726A* mutations.

One patient with concomitant FMF and IBD, both diagnosed before CNO, was classified in the markedly elevated APR group. In this patient, treatment was modified by switching between anti–IL-1, anti–IL-6 and anti-TNF agents because of persistent subclinical inflammation and disease activity. Among the remaining patients, FMF preceded CNO in three patients and followed CNO in eight, while IBD preceded CNO in two patients and developed after CNO in three.

One patient had concomitant uveitis and psoriasis, requiring switching between different anti-TNF agents according to disease activity. Among the remaining patients with uveitis, three received adalimumab, whereas one patient with isolated uveitis had a non-refractory course and was successfully treated with topical therapy and systemic corticosteroids without bDMARDs.

The comparison between patients with FMF and/or IBD (*n* = 15) and patients without any CNO-associated diseases (*n* = 29) revealed that haemoglobin and MCV were significantly lower, whereas platelet count, CRP and total number of bone lesions on WBMRI were significantly higher in patients with FMF and/or IBD. Further, when patients with other CNO-associated diseases (*n* = 7) and patients without any CNO-associated diseases (*n* = 29) compared, only total number of bone lesions on WBMRI was significantly higher in patients with any CNO-associated disease other than FMF and/or IBD (Table 3).

**Table 3:** Comparison of patients according to the type of CNO-associated disease.

Variables	No associated disease (N = 29)	FMF and/or IBD (N = 15)	Other associated diseases^#^ (N = 7)	p_1_-value*	p_2_-value**
**Haemoglobin [g/dL, median (IQR)]**	12.5 (12–13.1)	12.1 (10.9–13.4)	13.2 (12.1–13.5)	***0.031*** *^a^*	*0.719^a^*
**MCV [fL, median (IQR)]**	79.6 (76.6–82.8)	75.3 (71.5–77.9)	77.6 (76.2–78.7)	***0.009*** *^a^*	*0.208^a^*
**RBC count [x10^6^/mm^3^, median (IQR)]**	4.95 (4.42–5.28)	4.74 (4.53–5.18)	5.11 (4.90–5.41)	*0.746^a^*	*0.238^a^*
**WBC count [/mm^3^, median (IQR)]**	7940 (6790–9400)	8770 (7330–10800)	9900 (8580–11605	*0.223^a^*	*0.119^a^*
**PNL count [/mm^3^, median (IQR)]**	4470 (3660–5500)	4940 (3638–5848)	5700 (4895–7160)	*0.669^a^*	*0.168^a^*
**Lymphocyte count [/mm^3^, median (IQR)]**	2330 (1820–3010)	2590 (1950–3418)	2730 (2525–3350)	*0.445^a^*	*0.181^a^*
**Platelets [/mm^3^, median (IQR)]**	306000 (299000–414000)	441500 (384250–520500)	368000 (334000–475000)	***0.002*** *^a^*	*0.110^a^*
**MPV [fl, median (IQR)]**	9.6 (9–10.3)	9.65 (8.65–10.3)	9.1 (7.6–10.1)	*0.697^a^*	*0.357^a^*
**RDW [fl, median (IQR)]**	13.9 (13.1–14.7)	13.8 (13.4–14.7)	14.6 (13.9–15.4)	*0.542^a^*	*0.194^a^*
**CRP [mg/L, median (IQR)]**	5.1 (2.1–21.3)	20.6 (8.55–61.7)	10 (7.05–20.9)	***0.045*** *^a^*	*0.448^a^*
**ESR [mm/h, median (IQR)]**	17 (9–34)	25.5 (17.5–35.8)	21 (10–53)	*0.173^a^*	*0.645^a^*
**Ferritin [mg/dL, median (IQR)]**	20.8 (15–40.6)	22.3 (14.3–35.4)	25.8 (20.3–54.6)	*0.577^a^*	*0.422^a^*
**Total number of bone lesions [median (IQR)]**	5 (3–8)	11 (7–15)	8 (8–12)	***<0.001*** *^a^*	***0.011*** *^a^*

a Mann-Whitney *U* test.

# Other associated diseases (*n* = 7) included uveitis (*n* = 5), psoriasis (*n* = 2), and acnea (*n* = 3). The numbers are not mutually exclusive.

* p1-value represents the comparison between groups ‘patients without CNO-associated diseases’ and ‘patients with FMF and/or IBD’.

** p2-value represents the comparison between groups ‘patients without CNO-associated diseases’ and ‘patients with comorbidities other than FMF and IBD’.

CRP: C-reactive protein; CNO: chronic nonbacterial osteomyelitis; ESR: erythrocyte sedimentation rate; FMF: familial Mediterranean fever; IBD: inflammatory bowel disease; IQR: interquartile range; MCV: mean corpuscular volume; MPV: mean platelet volume; PNL: polymorphonuclear leukocytes; RBC: red blood cell; RDW: red cell distribution width; WBC: white blood cell.

## Discussion

In this historical cohort study, we evaluated the frequency of markedly elevated APRs (as defined by the 2025 ACR/EULAR paediatric CNO classification criteria) at the diagnosis of CNO and highlight differences in patient and disease characteristics between patients with and without markedly elevated APRs. Notably, markedly elevated APRs at diagnosis were observed in one-third of the patient population who exhibited lower haemoglobin but higher platelet and ferritin concentrations, more extensive involvement of CNO, particularly in clavicle, pelvis and sacroiliac joints, and a higher likelihood for CNO-associated diseases, including FMF and IBD. These findings suggest that marked elevation in APRs may be associated with a more severe inflammatory disease phenotype, although they are unfavourable for the classification of paediatric CNO.

Acute phase reactants are measured at presentation but their pattern in paediatric CNO is heterogeneous and cannot reliably rule in or out the disease. Many patients have only modest or no APR elevation, while those with higher values are more likely to have CNO mimickers, which lead lower classification scores in the 2025 ACR/EULAR paediatric CNO criteria [7]. The frequency of elevated APRs in paediatric CNO varies widely across studies, between 19% and 90% with most elevations being mild to moderate, reflecting the heterogeneity of the disease and differences in study populations and definitions [18]. In this study, although 63% of the patients had increased APRs, 31% had markedly elevated APRs, as defined by the CRP and ESR cut-offs in the classification criteria, which was associated with significantly lower total classification scores. The majority of these patients reached the threshold required for classification as CNO; however, ∼8% of the patients could not be classified as CNO because of high APRs. Overall, these results may indicate that markedly elevated APRs may lead to underclassification of patients with paediatric CNO, highlighting the need for cautious interpretation of APRs in clinical practice.

Limited data is available that investigates the association of elevated APRs with clinical findings of severe disease and greater imaging burden in paediatric CNO. A longitudinal study reported that an elevated ESR at diagnosis was related with higher disease activity over time and each mm/h elevation of ESR may raise the risk for severe disease by 3% [19]. Furthermore, increased APRs predicted total number of bone lesions [20]. High ESR at diagnosis was also associated with disease recurrence [21]. This study demonstrated that although markedly elevated APRs were not associated with differences in clinical findings, need for biologic therapy, or remission rates, these patients exhibited lower haemoglobin levels and higher platelet and ferritin concentrations, reflecting a more pronounced systemic inflammatory profile. In addition, they had significantly higher total number of bone lesions on WBMRI and more frequent involvement of the clavicle, pelvis and sacroiliac joints. Besides, a moderate correlation was observed between CRP and total number of MRI lesions. Collectively, these findings support that elevated APRs reflect increased inflammatory burden and disease extent in paediatric CNO. However, the presence of elevated APRs at baseline did not influence our treatment decisions, nor did it affect clinical or radiological remission rates. This finding suggests the effectiveness of the cDMARDs and bDMARDs used in our treatment approach.

Chronic nonbacterial osteomyelitis is frequently associated with other inflammatory diseases, most commonly including IBD and psoriasis, as well as FMF in Mediterranean populations [22–24]. The presence of CNO-associated diseases was related to the more extensive involvement of CNO and a higher need for biologics [23]. The prevalence of CNO-associated diseases was 43% in this study, while the most common diseases were FMF (22%), IBD (10%) and uveitis (10%). Patients with CNO-associated diseases had a higher number of bone lesions on WBMRI. Additionally, a diagnosis of FMF and/or IBD was related with significantly higher CRP levels and total number of bone lesions when compared with the patients without any associated diseases. Thus, CNO-associated diseases are common and are linked to more extensive disease and higher levels of APRs. These differences are particularly pronounced in patients with associated FMF and IBD. Thus, significant elevations in APRs may indicate the presence of CNO-associated diseases, particularly FMF and IBD, and warrant careful evaluation for these conditions. Coexisting conditions prior to the diagnosis of CNO, such as IBD, skin disorders and axial arthritis, have specific points in the recently developed EULAR/ACR classification criteria. Additionally, comorbid FMF should be considered in regions endemic to FMF.

The main limitations of this study include its retrospective design, single-centre setting and relatively modest sample size. Additionally, the limited geographic representation and high incidence of FMF in the Turkish population may affect the generalizability of our findings. Despite these limitations, the study offers valuable evidence on the relationship between elevated APRs, their impact on disease classification, disease phenotype and associated comorbidities in a well-characterized paediatric CNO cohort. Additionally, all patients included in the study underwent WBMRI or bone scintigraphy, allowing accurate determination of the number of bone lesions associated with CNO.

In conclusion, markedly elevated APRs are observed in a substantial subset of paediatric CNO patients that may cause classification limitations. These patients show more severe systemic inflammation, more extensive involvement, particularly in clavicle, pelvis and sacroiliac joints, and a higher likelihood of CNO-associated diseases such as FMF and IBD.

## Supplementary Material

keag495_Supplementary_Data

## Data Availability

The data that support the findings of this study are not publicly available but available on reasonable request from the corresponding author.
