# Supplementary material for: Acute phase reactants in paediatric chronic nonbacterial osteomyelitis: implications for diagnosis and correlation with disease phenotype
Source: Rheumatology (Oxford). 2026 Sep 9;65(9):keag495. doi: 10.1093/rheumatology/keag495 (PMC13615710; doi:10.1093/rheumatology/keag495)
Supplement: Supplementary file 1 [file keag495_supplementary_data.docx]

Supplementary material

**Supplementary Table S1:** Laboratory findings of the study groups at the diagnosis of chronic nonbacterial osteomyelitis

| **Variables** | All patients  (N=51) | Patients with normal-to-moderately elevated APRs  (N=35) | Patients with markedly elevated APRs  (N=16) | *p1-value** | Patients with normal APRs (N=19) | Patients with high APRs (N=32) | *p2-value*** |
| --- | --- | --- | --- | --- | --- | --- | --- |
| **Hemoglobin (g/dL, median (IQR))** | 12.3 (11.9-13.1) | 12.5 (12.1-13.3) | 12.1 (10.5-12.8) | ***0.049^a^*** | 12.5 (11.9-13.3) | 12.3 (11.4-12.8) | *0.413^a^* |
| **MCV (fL, median (IQR))** | 77.6 (75-81.2) | 78.4 (75.6-81.9) | 75.3 (71.5-79) | *0.094^a^* | 78.7 (76.4-82.8) | 77.2 (73.5-80.2) | *0.085^a^* |
| **RBC count (x10^6^/mm^3^, median (IQR))** | 4.9 (4.57-5.35) | 4.88 (4.55-5.3) | 5 (4.78-5.39) | *0.549^a^* | 4.65 (4.46-5.27) | 4.98 (4.66-5.39) | *0.126^a^* |
| **WBC count (/mm^3^, median (IQR))** | 8130 (7090-10485) | 8010 (6745-10485) | 8530 (8020-10513) | *0.273^a^* | 7220 (6050-10000) | 8660 (7930-10800) | ***0.025^a^*** |
| **PNL count (/mm^3^, median (IQR))** | 4910 (3680-5815) | 4770 (3740-5680) | 5485 (3638-6775) | *0.484^a^* | 4320 (3465-5125) | 5340 (4350-6475) | *0.055^a^* |
| **Lymphocyte count (/mm^3^, median (IQR))** | 2540 (1930-3215) | 2460 (1930-3040) | 2690 (2098-3238) | *0.784^a^* | 2330 (1820-3160) | 2685 (2155-3208) | *0.489^a^* |
| **Platelets (/mm^3^, median (IQR))** | 368000 (302000-456500) | 329000 (297000-436000) | 439000 (385500-542000) | ***0.003^a^*** | 339000 (279500-436000) | 395500 (303750-474750) | *0.072^a^* |
| **MPV (fL, median (IQR))** | 9.6 (8.95-10.2) | 9.7 (9.35-10.3) | 8.45 (7.67-9.27) | ***0.002^a^*** | 10 (9.5-10.7) | 9.15 (8.3-9.95) | ***0.010^a^*** |
| **RDW (fL, median (IQR))** | 14 (13.3-14.8) | 13.9 (13.3-14.6) | 14.5 (13.5-16.4) | *0.227^a^* | 13.7 (13.1-14.1) | 14.6 (13.4-15.1) | ***0.038^a^*** |
| **CRP (mg/L, median (IQR))** | 12 (2.7-29.3) | 5.1 (1.75-14.1) | 53.2 (38.9-76.1) | ***<0.001^a^*** | 1.9 (0.6-3.15) | 21.6 (13.9-52.8) | ***<0.001^a^*** |
| **ESR (mm/h, median (IQR))** | 21 (11-34.5) | 13 (8-22.5) | 51.5 (34.5-68.3) | ***<0.001^a^*** | 11 (6.5-16) | 33.5 (21-53) | ***<0.001^a^*** |
| **Ferritin (mg/dL, median (IQR))** | 22.6 (14.8-42.6) | 19.5 (14.8-27.9) | 71.5 (20.6-126) | ***0.041^a^*** | 19.4 (15.1-22.8) | 30.2 (13.4-79.6) | *0.078^a^* |

*^a^Mann-Whitney U-test*

*APR: Acute phase reactant, CRP: C-reactive protein, ESR: Erythrocyte sedimentation rate, IQR: Interquartile range, MCV: Mean corpuscular volume, MPV: Mean platelet volume, PNL: Polymorphonuclear leukocytes, RBC: Red blood cell, RDW: Red cell distribution width, WBC: White blood cell*

**p1-value represents the comparison between groups‘patients with normal-to-moderately elevated APRs’ and ‘patients with markedly elevated APRs’. Patients with ESR ≥60 mm/hour and/or CRP≥30 mg/L at the diagnosis were defined as the ‘patients with markedly elevated APRs’ according to the threshold values specified in the 2025 ACR/EULAR pediatric CNO classification criteria while patients below these thresholds were classified as ‘patients with normal-to-moderately elevated APRs’.*

***p2-value represents the comparison between groups ‘patients with normal APRs’ and ‘patients with high APRs’Any elevation in CRP and/or ESR at the diagnosis of CNO was defined as ‘patients with high APRs’ while others were ‘patients with normal APRs’.*
